# Tissue Turnover Rates and Isotopic Trophic Discrimination Factors in the Endothermic Teleost, Pacific Bluefin Tuna (*Thunnus orientalis*)

**DOI:** 10.1371/journal.pone.0049220

**Published:** 2012-11-07

**Authors:** Daniel J. Madigan, Steven Y. Litvin, Brian N. Popp, Aaron B. Carlisle, Charles J. Farwell, Barbara A. Block

**Affiliations:** 1 Tuna Research and Conservation Center, Hopkins Marine Station of Stanford University, Pacific Grove, California, United States of America; 2 Hopkins Marine Station of Stanford University, Pacific Grove, California, United States of America; 3 Department of Geology and Geophysics, University of Hawai'i at Manoa, Honolulu, Hawai'i, United States of America; 4 Monterey Bay Aquarium, Monterey, California, United States of America; Universitat de Barcelona, Spain

## Abstract

Stable isotope analysis (SIA) of highly migratory marine pelagic animals can improve understanding of their migratory patterns and trophic ecology. However, accurate interpretation of isotopic analyses relies on knowledge of isotope turnover rates and tissue-diet isotope discrimination factors. Laboratory-derived turnover rates and discrimination factors have been difficult to obtain due to the challenges of maintaining these species in captivity. We conducted a study to determine tissue- (white muscle and liver) and isotope- (nitrogen and carbon) specific turnover rates and trophic discrimination factors (TDFs) using archived tissues from captive Pacific bluefin tuna (PBFT), *Thunnus orientalis*, 1–2914 days after a diet shift in captivity. Half-life values for ^15^N turnover in white muscle and liver were 167 and 86 days, and for ^13^C were 255 and 162 days, respectively. TDFs for white muscle and liver were 1.9 and 1.1‰ for *δ* ^15^N and 1.8 and 1.2‰ for *δ* ^13^C, respectively. Our results demonstrate that turnover of ^15^N and ^13^C in bluefin tuna tissues is well described by a single compartment first-order kinetics model. We report variability in turnover rates between tissue types and their isotope dynamics, and hypothesize that metabolic processes play a large role in turnover of nitrogen and carbon in PBFT white muscle and liver tissues. ^15^N in white muscle tissue showed the most predictable change with diet over time, suggesting that white muscle *δ* ^15^N data may provide the most reliable inferences for diet and migration studies using stable isotopes in wild fish. These results allow more accurate interpretation of field data and dramatically improve our ability to use stable isotope data from wild tunas to better understand their migration patterns and trophic ecology.

## Introduction

Stable isotope analysis (SIA) is a popular ecological tool that is increasingly used to address a variety of topics, including trophic ecology and animal migration. Researchers have used SIA to examine nutrient flow, trophic dynamics, and structural changes in ecosystems [1], [2], [3], [4], [5], and isotope studies have also elucidated the origins, variation, and timing of movements of migratory animals [6], [7], [8]. However, the number of stable isotope studies of natural systems far outnumbers laboratory-based studies which can be necessary to validate and interpret results [9]. Foreseeing the growth of SIA studies, Gannes et al. [9] called for more laboratory experiments, and though the number of studies has increased, additional controlled laboratory studies are needed for the many ecosystems, food webs, species and tissues subjected to SIA [10]. Specifically, the dynamics of consumer-prey isotope discrimination and of tissue-specific turnover rates of carbon and nitrogen are required by researchers to correctly interpret SIA data from wild organisms [9].

SIA uses the ratio of a heavier, less common isotope to a lighter, more common isotope, most often the ratios of ^15^N/^14^N (*δ* ^15^N) and ^13^C/^12^C (*δ* ^13^C) in ecological studies using plant and animal tissues. Carbon and nitrogen isotopes are fractionated (i.e., *δ*-values increase) between trophic levels in food webs; however the trophic increase of ^13^C has been shown to be lower than that of ^15^N across various taxa [11]. Due to this minimal fractionation in food webs, *δ* ^13^C values are often used to estimate carbon source inputs to consumer diets (e.g., C3 or C4 plants, macroalgae or phytoplankton) when *δ* ^13^C values are different at the producer level. In contrast, *δ* ^15^N values tend to increase more with each trophic step, and accordingly are often used to estimate the trophic level of organisms within food webs.

More complex tools that use SIA (e.g., mixing models [12], isotopic clocks [13]) require accurate tissue-specific trophic discrimination factors (TDFs; the difference between the *δ*-values of a consumer's tissues and its diet) and isotopic turnover rates to make reliable estimates of consumer foraging and the origin and timing of migrations. Isotopic turnover is defined as the time it takes for a given consumer tissue to reflect the isotopic composition of food resources, and is the result of both tissue growth and tissue replacement [14], [15], [16]. Turnover rates can be measured by monitoring tissue isotope values over time until steady-state is established between tissue and diet. A function is then fitted to the change in isotope composition over time, and a half-life for that tissue and isotope can be calculated [14], [15], [16]. Tissue turnover rates for blood, liver, skeletal muscle, and other tissues have been described in several taxa [14], [17], [18], [19], [20]. Turnover rates depend on metabolic processes within animal tissues, mass, and growth, all of which vary with ontogeny, across taxa, and across tissue types. Using inappropriate TDF and turnover values can lead to erroneous interpretation of stable isotope data [12], [21]. Accurate isotopic turnover and TDF values are highly useful for improving isotope models, such as mixing models [12], [21] and isotopic clock approaches [13], [22]. Thus lab-controlled studies of stable isotope dynamics in animal tissues greatly improve our capacity to use isotopic data to study the natural history (e.g., diet and migrations) of the organisms of interest.

Pelagic animals, including seabirds [23], [24], pinnipeds [25], sharks [8], [26], [27], and teleosts [28], have been increasingly studied using SIA. Due to the oceanic lifestyle of these animals, studies of their ecology and migration patterns have historically been difficult. Feeding ecology studies have relied on traditional gut content analyses (GCA). While providing species-specific diet information that SIA cannot provide, GCA often provides only a snapshot of predator diet [29]. While long-term, comprehensive studies using GCA are possible, they are extremely time- and labor-intensive. The movements of pelagic organisms have historically been difficult to study, though electronic tagging has significantly increased our understanding of the movements of these highly migratory pelagic species [30]. However, electronic tags are expensive, deployments are challenging to execute, and only in rare instances can the electronic tag provide data regarding foraging or diet information, though feeding has been demonstrated using tags in wild tunas, pinnipeds, and sharks [31], [32], [33]. Furthermore, electronic tagging only provides movement data while the tag is functioning on the animal and provides no information on retrospective movements. SIA serves as a powerful complement to electronic tagging and GCA, allowing the capacity to track large scale oceanic movements and diet using carbon and nitrogen isotope values. Few validation studies on stable isotope dynamics exist in large, predatory pelagic teleosts due to the difficulty of holding large pelagics in captivity. Thus, laboratory-based studies of stable isotope dynamics in pelagic fishes are conspicuously absent, yet necessary, in order to effectively apply SIA to study the ecology of large pelagic species and ecosystems.

The long-term holding of captive Pacific bluefin tuna, *Thunnus orientalis*, (PBFT) at the Tuna Research and Conservation Center (TRCC) and Monterey Bay Aquarium (MBA) [34] provides a unique opportunity to track the changes in isotopic composition of multiple tissues over a long time period after collection from the wild and a change to a controlled diet with a different isotopic composition in a temperature-controlled tank setting. PBFT used in this study were kept in captivity for up to 2914 days, providing the longest dataset available for a large pelagic fish fed a controlled diet. We aimed to 1) calculate turnover rates in PBFT white muscle and liver, two commonly used tissues in isotope studies and 2) calculate the TDFs of PBFT from animals in which tissue isotopic composition had reached steady-state with the controlled diet. In addition we estimated the relative importance of growth vs. metabolism in tissue turnover rates. Together TDF values and turnover rates can be applied to data from wild tunas to study aspects of their feeding ecology and migration with a precision that has thus far not been possible.

## Results

Both white muscle (WM) and liver (LIV) tissues reached asymptotic values, representing steady-state with diet in time- and growth-based models. This allowed for reliable calculation of tissue turnover rates and trophic discrimination factors for fish held in captivity from 1–2914 days (Table 1). A single-compartment model with first-order kinetics adequately described changes in carbon and nitrogen isotopic compositions of liver and white muscle tissues during the early stages (0–725 days) of the study. Application of the reaction progress variable (RPV) showed no evidence of multiple turnover pools (Fig. S1), and the RPV model is sensitive to the steady-state isotopic composition [35], [36], thus results from the latter portions of this study were difficult to evaluate using the RPV approach. Exponential functions thus provided the best fit for turnover rates of C and N in both white muscle and liver.

**Table 1:** Mean stable isotope values and time in captivity for all Pacific bluefin tuna (*Thunnus orientalis*) used in this study. *δ* ^13^C′ values are arithmetically-corrected for lipid content based on tissue- and species-specific (*Thunnus thynnus*) algorithms from Logan et al. [51].

n	Time in captivity (d)	ΔMass (SD) (kg)	WM *δ* ^15^N (SD)	WM *δ* ^13^C′ (SD)	LIV *δ* ^15^N (SD)	LIV *δ* ^13^C′ (SD)
3	0	—	11.8 (0.2)	−18.9 (0.4)	11.6 (0.8)	−18.0 (1.3)
18	1–99	0.79 (0.93)	12.9 (0.7)	−17.7 (0.6)	13.8 (0.7)	−17.5 (0.8)
8	100–199	1.77 (1.27)	13.5 (0.8)	−17.6 (0.5)	14.0 (0.7)	−17.7 (0.5)
10	200–299	3.47 (1.13)	14.3 (0.3)	−17.6 (0.3)	14.4 (0.7)	−17.4 (0.4)
7	300–399	9.17 (2.38)	14.8 (0.9)	−17.0 (0.5)	14.6 (0.4)	−16.7 (0.5)
3	400–499	6.37 (0.95)	15.2 (0.3)	−16.1 (0.4)	15.2 (0.7)	−16.3 (0.4)
5	500–599	7.43 (1.56)	15.3 (0.2)	−16.9 (0.3)	15.0 (0.6)	−16.8 (0.5)
4	600–999	38.65 (47.96)	15.4 (0.3)	−16.0 (0.3)	15.1 (0.3)	−16.1 (0.5)
10	1000–1999	73.88 (42.75)	15.7 (0.3)	−16.3 (0.4)	15.2 (0.8)	−16.0 (1.5)
2	2000–2914	167.54 (43.73)	16.1 (0.3)	−16.4 (0.4)	14.6 (—)	−15.1 (—)

Isotope values and change in mass (Δmass) reported as mean (SD).

### Time-based *δ* ^13^C and *δ* ^15^N turnover

**Figure 1:**
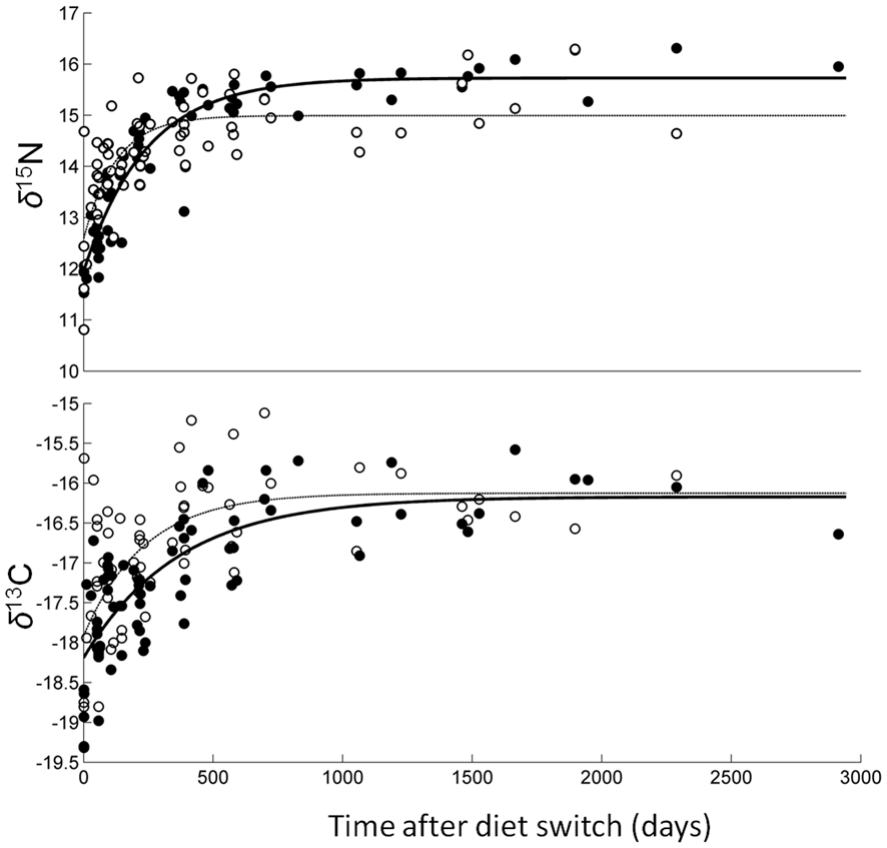
Isotopic change over time in white muscle and liver tissues in captive Pacific bluefin tuna (*Thunnus orientalis*). *δ* ^15^N and *δ* ^13^C values in Pacific bluefin tuna white muscle (WM; filled circles) and liver (LIV; open circles) are shown as a function of time (days) after change to isotopically distinct captive diet. Lines represent time-based exponential model fits for WM (solid line) and LIV (thin dotted line).

Turnover, based on changes in *δ* ^13^C and *δ* ^15^N values, was evident in the early stages (0–725 days) of the study in both WM and LIV tissues, after which steady-state was reached for both isotope values in each tissue (Fig. 1). Liver tissue turnover was faster for both *δ* ^13^C and *δ* ^15^N (*t*_0.5_ = 162 and 86 days, respectively) than turnover in WM (*t*_0.5_ = 255 and 167 days; Table 2). Estimated *t*_0.5_ for carbon turnover in WM based on allometric scaling of isotopic turnover rates in fish [37] was 183±42 days, and our calculated value (255 days) was within the 95% confidence interval of this estimate.

**Table 2:** Parameter estimates and 95% confidence intervals for time-based exponential fit models for each tissue (WM or LIV) and isotope (*δ* ^15^N or *δ* ^13^C) in Pacific bluefin tuna (*Thunnus orientalis*).

		Parameter (95% CI)					
Tissue	Isotope	*a*	*b*	*λ*	r^2^	*t*_0.5_ (d) (95% CI)	*t*_0.95_ (d)
WM	*δ* ^15^N	15.73 (15.46, 15.99)	−3.78 (−4.15, −3.41)	−0.0042 (−0.0052, −0.0032)	0.86	167 (134, 222)	721
WM	*δ* ^13^C	−16.17(−16.52, −15.83)	−2.02 (−2.41, −1.63)	−0.00272 (−0.00413, −.00130)	0.61	255 (168, 532)	1103
LIV	*δ* ^15^N	14.99 (14.68, 15.31)	−2.41 (−3.00, −1.82)	−0.00805 (−0.0125, −0.00364)	0.53	86 (56, 190)	372
LIV	*δ* ^13^C	−16.13 (−16.56, −15.69)	−1.79 (−2.35, −1.23)	−0.00428 (−0.00774, −0.000816)	0.39	162 (90, 850)	701

Estimated half-life (*t*_0.5_) and time for 95% isotope turnover (*t*_0.95_) is shown for each tissue and isotope.

In general, exponential model fits were better for WM tissue than for liver and for *δ* ^15^N than for *δ* ^13^C values, resulting in narrowest 95% CI estimates for *t*_0.5_ in WM *δ* ^15^N (134–222 d) and broadest 95% CI estimates for LIV *δ* ^13^C (90–850 d; Table 2). Using 95% turnover as the cutoff for steady-state isotopic conditions with diet (i.e. the time needed for *δ* ^13^C and *δ* ^15^N values in WM and LIV to accurately represent recent dietary inputs), liver *δ* ^15^N reached steady-state with diet first (*t*_0.95_ = 372 days) and white muscle *δ* ^13^C last (*t*_0.95_ = 1103 days).

### Tissue-specific TDF

Fish at steady-state with diet *δ* ^13^C and *δ* ^15^N values allowed for calculation of TDF values for *δ* ^13^C and *δ* ^15^N in WM and LIV tissues of PBFT. Sample size for TDF calculations ranged from n = 10 (LIV *δ* ^13^C′) to n = 24 (LIV *δ* ^15^N) (Table 3). Mass-weighted feed mean *δ* ^13^C and *δ* ^15^N values, hereafter reported as mean ± SD (*δ* ^13^C: −17.4±0.3; *δ* ^15^N: 13.9±0.7) were higher than initial PBFT white muscle values (*δ* ^13^C: −18.0±0.2; *δ* ^15^N: 11.8±0.2) and lower than mean steady-state values of *δ* ^13^C and *δ* ^15^N in WM and LIV (Table 3). TDF values for *δ* ^13^C′ are for arithmetically lipid-corrected bulk *δ* ^13^C values for both consumer (PBFT) and food. TDF values for *δ* ^13^C′ and *δ* ^15^N in WM were 1.8±0.3‰ and 1.9±0.4‰, respectively; TDF values for *δ* ^13^C′ and *δ* ^15^N in LIV were 1.2±0.3‰ and 1.1±0.6‰, respectively (Table 3).

**Table 3:** Table of mean *δ* ^15^N, *δ* ^13^C (bulk), *δ* ^13^C′ (arithmetically lipid-corrected [51]), and bulk C∶N ratio values of Pacific bluefin tuna (*Thunnus orientalis*) tissues (WM and LIV), captive feed (white muscle WM or whole Wh), and calculated tissue-specific TDF values for white muscle and liver (± SD).

Group	Isotope	Tissue	n	Mean (SD)	C∶N (SD) (mass)			
**Feed**								
Sardine	*δ* ^13^C′	WM	13	−17.0 (0.5)	3.2 (0.1)			
	*δ* ^13^C	WM	13	−17.3 (0.5)	3.2 (0.1)			
	*δ* ^15^N	WM	13	13.3 (0.8)	3.2 (0.1)			
Squid	*δ* ^13^C′	WM	12	−17.7 (0.2)	3.5 (0.1)			
	*δ* ^13^C	WM	12	−18.4 (0.2)	3.5 (0.1)			
	*δ* ^15^N	WM	12	14.5 (0.7)	3.5 (0.1)			
Supplement	*δ* ^13^C′	Wh	2	−17.2 (0.1)	4.2 (0)			
	*δ* ^13^C	Wh	2	−20.2 (0.0)	4.2 (0)			
	*δ* ^15^N	Wh	2	11.6 (0.1)	4.2 (0)			
**Feed mean**	*δ* ^13^C′	—	27	−17.4 (0.3)	—			
**(weighted by mass)**	*δ* ^13^C	—	27	−18.2 (0.3)	—			
	*δ* ^15^N	—	27	13.9 (0.7)	—	**TDF**		
**Consumer**						**Mean**	**SD**	**Time in capt.**
PBFT	*δ* ^13^C′	WM	10	−15.6 (0.3)	4.9 (1.8)	1.8	0.3	>1103 d
PBFT	*δ* ^13^C	WM	10	−17.7 (1.8)	4.9 (1.8)	0.5	1.8	>1103 d
PBFT	*δ* ^15^N	WM	14	15.7 (0.4)	4.9 (2.0)	1.9	0.4	>721 d
PBFT	*δ* ^13^C′	LIV	10	−16.2 (0.5)	7.6 (3.4)	1.2	0.3	>701 d
PBFT	*δ* ^13^C	LIV	11	−19.8 (2.0)	7.6 (3.4)	−1.6	2.0	>701 d
PBFT	*δ* ^15^N	LIV	24	15.1 (0.6)	6.2 (2.6)	1.1	0.6	>372 d

Fish used for TDF calculations were in captivity for a time period that allowed for at least 95% isotopic turnover for each tissue and isotope (see Table 2).

### Growth

**Figure 2:**
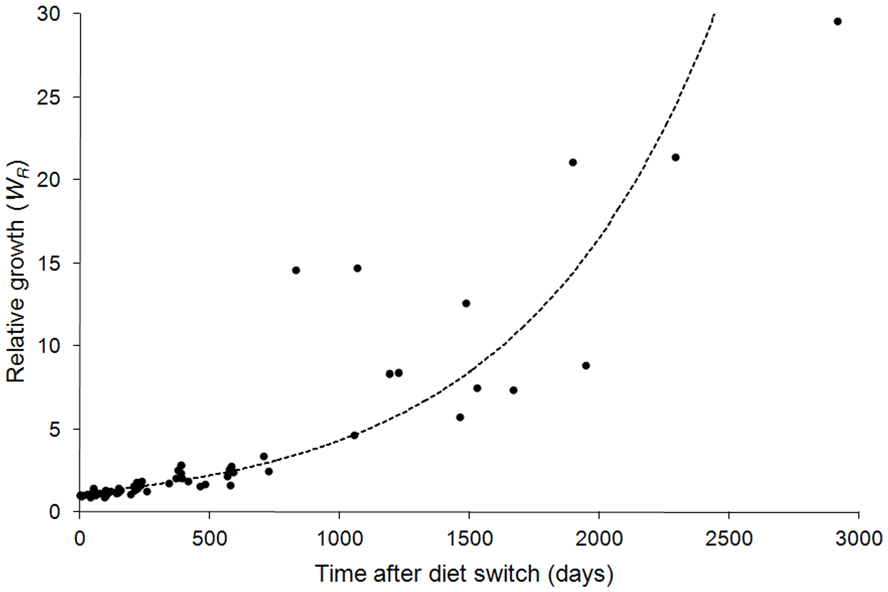
Relative growth (*W_R_*) for individual Pacific bluefin tuna over time (days) in captivity. Dashed line represents exponential model fit to data (r^2^ = 0.87).

**Figure 3:**
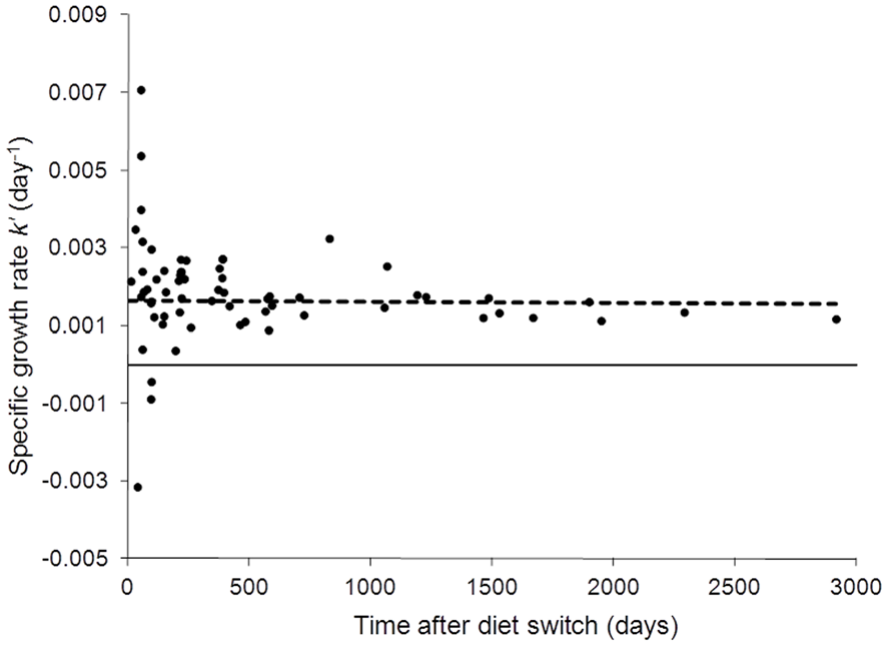
Specific growth rates (*k*′) for Pacific bluefin tuna in captivity. Growth rates were calculated from relative growth (W_R_) and time in captivity (*t*). Dashed line represents linear fit to data. Estimates of group growth rates (y-intercept of linear fit and mean *k*′) are the same (*k*′ = 0.0016).

PBFT held in captivity on a controlled diet showed substantial growth in TRCC and MBA tanks, with one individual increasing in mass by a factor of ∼30 (Fig. 2). Growth of captive PBFT was linear from 0–725 days, and then became exponential between 725–2914 days. An exponential equation best fit the overall growth data (dashed line, Fig. 2; r^2^ = 0.87). The switch from linear to exponential growth was most likely a result of moving tuna from TRCC to MBA tanks, where tank volume increased and feed ratios remained the same but quantity increased (C. Farwell, pers. comm.). Specific growth rates (*k′*) were most variable for fish early in the experiment, ranging from 0.0071 to −0.0031 (Fig. 3). Only three fish showed negative *k′* values, which may have been a result of inadequate feeding early in captivity. Growth rates (*k′*) were more variable in the early stages of the experiment (0–800 d), then variability decreased throughout the course of the experiment (Fig. 3). Linear fit to *k′* data (dashed line, Fig. 3) had a slope near zero (−3.0×10^−8^) indicating a negligible change in *k′* over time in captivity. Overall *k′* can be estimated by the y-intercept of the linear fit to *k′* data or mean *k′*; these values were the same (*k′* = 0.0016).

### Growth-based *δ* ^13^C and *δ* ^15^N turnover

**Figure 4:**
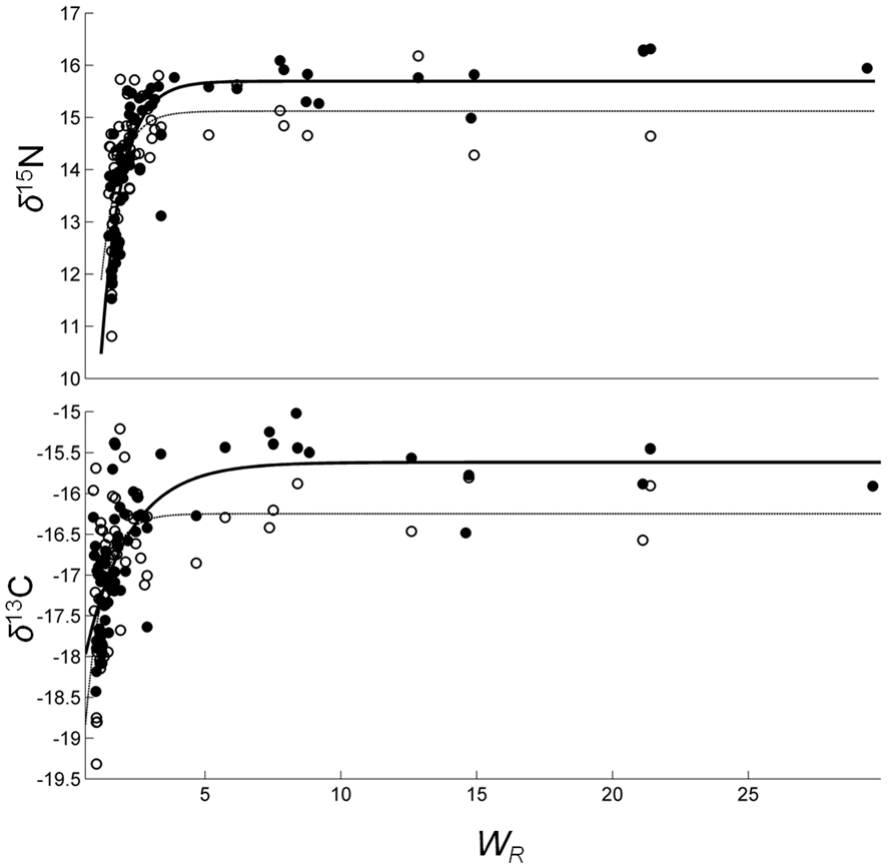
Isotopic change with growth in white muscle and liver tissues in captive Pacific bluefin tuna (*Thunnus orientalis*). *δ* ^15^N and *δ* ^13^C values in Pacific bluefin tuna white muscle (WM; filled circles) and liver (LIV; open circles) are shown as a function of relative growth (*W_R_*) after switch to isotopically distinct captive diet. Lines represent time-based exponential model fits for WM (solid line) and LIV (thin dotted line).

Change in *δ* ^13^C and *δ* ^15^N values in white muscle and liver was well represented by growth-based models, although fits (*r* ^2^ values) were slightly lower than those from time-based models (Tables 2, 4). Relative growth-based turnover was faster in liver than in white muscle for both *δ* ^13^C and *δ* ^15^N values (Fig. 4). The growth-based half-life was shortest for liver *δ* ^13^C (1.56) and highest for WM *δ* ^13^C (3.09) (Table 4). Growth (increase in mass) necessary for 50% turnover of nitrogen in white muscle and liver was 72% and 59%, respectively, and 56% and for carbon in liver. A higher gain in mass (209%) was necessary for 50% turnover of carbon in white muscle (Table 4).

**Table 4:** Parameter estimates and 95% confidence intervals for relative growth-based (*W_R_*) exponential fit models for each tissue (WM or LIV) and isotope (*δ* ^15^N or *δ* ^13^C) in Pacific bluefin tuna (*Thunnus orientalis*).

		Parameter (95% CI)				
Tissue	Isotope	*a*	*b*	*c*	r^2^	*G*_0.5_
WM	*δ* ^15^N	15.7 (15.33, 16.06)	−11.26 (−16.68, −5.84)	−1.28 (−1.756, −0.8032)	0.72	1.72
WM	*δ* ^13^C	−15.62 (−15.94, −15.29	−3.402 (−4.623, −2.181)	−0.615 (−0.9454, −.2837)	0.58	3.09
LIV	*δ* ^15^N	15.12 (14.65, 15.6)	−7.933 (−17.0, 1.132)	−1.49 (−2.669, −0.3107)	0.39	1.59
LIV	*δ* ^13^C	−16.25 (−16.69, −15.81)	−6.604 (−16.1, 2.894)	−1.557 (−3.039, −0.0739)	0.30	1.56

Estimated growth-based half-life (*G*_0.5_) is shown for each tissue and isotope.

### Contributions to turnover of growth and metabolic processes

Routine metabolism, as the sum of all anabolic and catabolic processes, contributes to both components of isotope turnover in tuna tissues: new tissue growth, and all other metabolic processes [15], [16]. Studies of isotopic turnover in fish often discern the effects of growth from metabolism [18]. Thus all metabolic processes, excluding growth, are hereafter referred to simply as ‘metabolic processes’ or ‘metabolism’. Overall estimates of proportion of turnover due to growth or metabolism for all tunas varied by tissue and by isotope (Table 5). In liver, metabolic processes contributed more to isotope turnover than in muscle, and more to turnover of nitrogen (85%) than of carbon (63%) (Table 5). Metabolic processes also contributed significantly to isotope turnover in muscle, accounting for 62% of WM nitrogen turnover and 41% of WM carbon turnover. Growth accounted for the majority of isotope turnover in only one isotope and tissue (WM carbon, 59%; Table 5).

**Table 5:** Parameter estimates and 95% confidence intervals for metabolic constant (*m*) from time-based exponential fits to *δ* ^15^N and *δ* ^13^C data for Pacific bluefin tuna (*Thunnus orientalis*) white muscle and liver.

		Parameter			
Tissue	Isotope	*k′*	*m* (95% CI)	*P_g_* (95% CI)	*P_m_* (95% CI)
WM	*δ* ^15^N	0.0016	0.00256 (0.00152, 0.00359)	0.38 (0.31, 0.51)	0.62 (0.49, 0.69)
WM	*δ* ^13^C	0.0016	0.00112 (0.00030, 0.00253)	0.59 (0.39, 0.84)	0.41 (0.16, 0.61)
LIV	*δ* ^15^N	0.0016	0.00942 (0.00546, 0.01337)	0.15 (0.11, 0.23)	0.85 (0.77, 0.89)
LIV	*δ* ^13^C	0.0016	0.00267 (0.00078, 0.00613)	0.37 (0.21, 0.67)	0.63 (0.33, 0.79)

Estimates and 95% confidence intervals of proportion of turnover due to growth (*P_g_*) and metabolism (*P_m_*) is shown for each tissue and isotope.

### Comparison of captive and wild data

**Figure 5:**
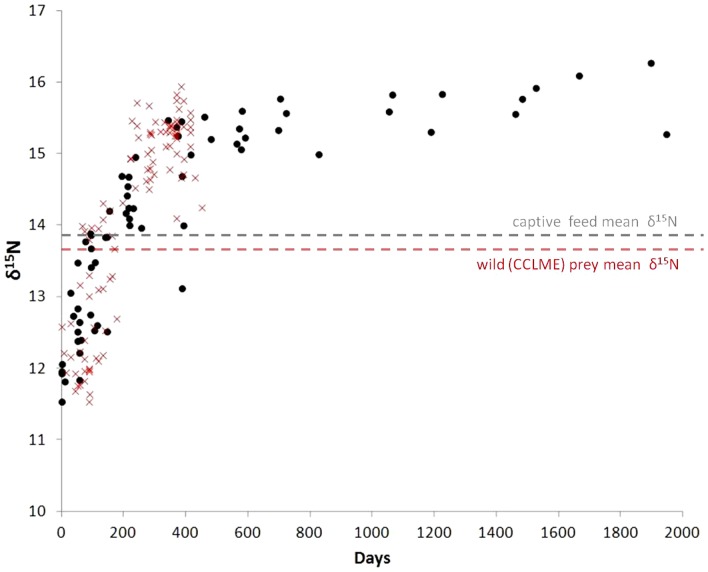
Change in ^15^N with time in captive and wild Pacific bluefin tuna (*Thunnus orientalis*). *δ* ^15^N values in captive (filled circles) and wild (red x's) Pacific bluefin tuna white muscle. Time (days) for wild fish represents estimated residency time in the California Current Large Marine Ecosystem, and was estimated from fish size [82], using the smallest sampled individual (61.6 cm) to approximate starting value for *t* (*t* = 0 days). Dashed lines show weighted mean *δ* ^15^N values for captive food (grey) and wild prey (red).

Data from wild Pacific bluefin tuna showed similar turnover of ^15^N to captive fish (Fig. 5). Samples from large wild bluefin tuna were not available, so we were unable to assess whether wild fish of larger sizes had reached steady-state with local prey. However the largest wild fish (which had the longest estimated residency times in the CCLME) had reached *δ* ^15^N values of 15–16‰ in white muscle tissue, similar to captive fish that had reached steady-state in captivity (Fig. 5).

## Discussion

Pacific bluefin tuna that are captured in the wild and then transported and held in captivity provide excellent subjects for validation experiments. We used archived tissue samples from Pacific bluefin tuna held in captivity for a wide range of time to design an experimental framework from which isotopic turnover rates and trophic discrimination factors can be accurately estimated. These data reveal several important aspects of isotope turnover and trophic discrimination in growing endothermic fish. White muscle and nitrogen showed more predictable turnover dynamics and better model fits than liver and carbon. The duration of time in captivity required for tissues to reach steady-state (95% turnover) with diet (fastest: LIV ^15^N = 372 days; slowest: WM ^13^C = 1103 days) demonstrates that isotope turnover experiments in fish may need to exceed several years to adequately represent full turnover of *δ* ^13^C and *δ* ^15^N values in certain tissues. Finally, these results suggest that metabolic processes may contribute more to isotopic turnover, particularly in muscle tissue, in Pacific bluefin tuna, an endothermic pelagic fish, relative to other fish species.

### Turnover in tissues

Overall, our tissue-specific turnover rates were lower (i.e. tissue turnover took longer) than values reported for mammals and birds [16], [38] and some ectothermic fish [39]. Body temperatures are significantly lower in bluefin tuna in comparison to birds and mammals (20–25°C for bluefin and 37–42°C for mammals and birds). However, our turnover rates were similar to values reported in leopard sharks by Kim et al. [40], where muscle tissue *δ* ^13^C and *δ* ^15^N values took several hundred days to reach steady-state and study animals were large (1–5 kg) compared to fish in most previous studies [37]. Weidel et al. [37] found an allometric relationship between fish size and isotope turnover; thus tissues in larger fish would take longer to reflect a diet switch. We estimated a white muscle carbon turnover half-life of 184±42 days in Pacific bluefin tuna based on the allometric relationship in Weidel et al. [37]. While application of large fish to this equation extrapolates far beyond the fish sizes used in the study [37], it does indicate that fish size can greatly affect turnover rates and may explain the relatively slow turnover times in the relatively large Pacific bluefin tuna. Allometric scaling of turnover rate with body size in white sharks, for example, led to extremely long estimates of carbon turnover time in white muscle (t_1/2_ = 394±42 days) [8]. Isotopic turnover rates have also been shown to be positively correlated with metabolic rate, at least in mammals [16]. As regional endotherms, or ‘heterotherms’ [41], Pacific bluefin tuna have lower metabolic rates than birds and mammals [42]. The larger size of the tuna in this study and the leopard sharks in Kim et al. [40], compared to fish in other studies of isotopic turnover, along with lower metabolic rates in fish (including regionally endothermic fish [41]), compared to birds and mammals, may provide the basis for the relatively long turnover times we observed. Further studies on tunas and other fish will help elucidate the relative importance of animal size and metabolic rate on isotopic turnover in various tissues.

Turnover rates of carbon and nitrogen were higher in liver tissue than in white muscle, which is consistent with results found in most other fish [20] although some studies found no differences between these tissues [14]. This has generally been attributed to the higher metabolic activity of liver tissue, in which protein synthesis and degradation can be much faster than in skeletal muscle tissue [18], [43], [44], [45]. Liver has also been shown in other studies to be much more variable in isotopic values than muscle [46], [47]. Speculative causes of high variability in liver are differences in amino acid composition of liver versus white muscle [46], [47] and higher metabolic activity in liver tissue [46]. Carbon turnover was slower than nitrogen turnover in both liver and white muscle tissues, though the opposite result has been found in some studies in fish [18], [48]. Differences in proportional contribution of metabolism versus growth to turnover have been proposed as the driving force between turnover rate differences between tissues and isotopes [18], with more metabolically active tissues showing higher turnover rates. Results here support this relationship, as the tissue (liver) and isotope (^15^N) with higher proportion of turnover attributable to metabolism (*P_m_*) (Table 5) both showed higher turnover rates (Table 2) than muscle and ^13^C, respectively. Thus the different proportional impacts of metabolism or growth on tissue turnover may determine which tissues and isotopes turn over faster in a given fish species [18].

*δ* ^13^C′ values were more variable in white muscle and liver tissues with correspondingly low correlation coefficients for exponential model fits of turnover. Since this study was not longitudinal, and we sampled unique individuals for each data point, individual variation could have led to the variability seen in *δ* ^13^C values (and *δ* ^15^N values as well). Individual variation has shown to be a potentially important factor in controlled studies of isotope discrimination and turnover [49]. Carbon isotope values of tissues can also be confounded by variable lipid content, with higher lipid in tissues resulting in lower *δ* ^13^C values due to the low ^13^C content of lipids relative to protein and carbohydrates [50], [51], [52]. Two methods that have emerged for estimating the effects of lipid content on *δ* ^13^C values are chemical extraction and arithmetic correction based on C∶N ratio, which can be used as a proxy for lipid content [50], [51], [52]. Our values were arithmetically corrected based on lipid-correction algorithms derived from Atlantic bluefin tuna, *Thunnus thynnus* [51]. Arithmetic corrections have been well supported by several studies, but lipids will likely continue to be a source of variation in *δ* ^13^C values in any lipid-rich tissues, whether they are chemically lipid-extracted or arithmetically-corrected. Liver tissues had higher lipid concentrations and C∶N ratios than white muscle tissue (Table S1) which may have contributed to the higher variability and poorer model fits for *δ* ^13^C′ in liver (Fig. 1; Table 2).

### Trophic discrimination factors (TDF)

Trophic discrimination factors (TDFs) reported here are the first for pelagic teleosts in which at least 95% turnover for both *δ* ^13^C and *δ* ^15^N in liver and white muscle was demonstrated and study animals have been fed a mixed diet (resembling natural food habits). Varela et al. [53] report TDF values for Atlantic bluefin tuna (*Thunnus thynnus*) reared on a captive diet for five months. Their Δ^15^N estimate (1.6‰) was similar to ours here (1.9‰), though their reported Δ^13^C value (−0.2‰) was lower [53] and suggests that PBFT will have lower *δ* ^13^C values than their diet. However, isotopic steady-state between tunas and diet was not demonstrated in this study and reliability of turnover rate estimates is highly dependent on tissues reaching steady-state with diet, or an asymptotic isotopic value [10]. The short rearing time (∼5 months, or 150 days) in the study by Varela et al. [53] was likely insufficient for captive tunas to reach steady-state, based on turnover rates reported here. In this study, ^15^N TDF for white muscle (Δ^15^N_WM_: 1.9±0.4) fell within the lower, but wide, range of Δ^15^N values reported for a variety of taxa (∼−1‰–6‰) [54] and the range reported in a review for WM Δ^15^N of 22 fish species (−1‰–5.6‰) [55]. However Caut et al. [55] reported an inverse relationship between Δ^15^N and *δ* ^15^N food value and proposed empirical algorithms to calculate ‘Diet-Dependent Discrimination Factors’ (DDDFs). We calculated a TDF estimate of 1.9‰ using the DDDF algorithm for fish WM [55], the same estimate as our experimental value of 1.9‰. This suggests that the high *δ* ^15^N value of tuna feed may impact our calculated TDFs, and that our experimentally-derived Δ^15^N values correlate with the TDFs for many fish species in Caut et al. [55]. The isotopic values of prey here are consistent with the diet of wild tuna in the California Current Large Marine Ecosystem (CCLME), which feed on higher trophic levels than fish in most other turnover studies using isotopes as tracers and thus feed on organisms with generally high *δ* ^15^N values [5], [56].

Our TDF values for carbon in both white muscle and liver fit within ranges found in previous studies, though Δ^13^C values (WM 1.8‰; LIV 1.2‰) were high relative to the most commonly referenced Δ^13^C ranges of 0–1‰ [11]. However it has been demonstrated that these values are highly taxa-, tissue-, and diet-dependent, and our values fall well within the range reported for 41 fish studies (−0.8–3.7‰) [55]. Our results show that TDF for ^15^N is lower and TDF for ^13^C is higher than in other taxa [11], which suggests that traditional utilizations of *δ* ^15^N values for trophic estimations and *δ* ^13^C values for food web sourcing may not be appropriate for PBFT. Liver TDF values were lower and TDF for liver nitrogen more highly variable, suggesting that while liver can be useful for making inferences about diet on shorter timeframes due to faster turnover, liver isotope values and TDFs should be used with caution due to their high variability.

Some mixing models (e.g. MixSir [12]) take TDF error into account, but TDF is one of the most influential factors on mixing model results [12]. The most useful application of the TDFs reported here will be for trophic assessments of tuna that are complementary to traditional dietary analyses, which can under-represent prey, particularly prey that are quickly digested or do not contain hard parts [29]. Such approaches have been put to use in the California Current Large Marine Ecosystem (CCLME) [5] and the Mediterranean [57] where they revealed high consumption of krill and gelatinous salps, respectively. TDFs reported here will support similar studies of tuna feeding habits, particularly using white muscle (which was least variable). Liver tissue, which turns over more rapidly, will provide more recent insight into diet. However, based on the variability of liver isotope values reported here, we recommend the use of liver TDF values in conjunction with other tissues, particularly WM, when calculating dietary reconstructions of wild tuna. In addition, due to the long turnover times reported here, trophic studies using SIA will benefit from complementary movement data (e.g. from electronic tags) to assess whether study animals have likely been feeding on local prey baselines for sufficiently long periods to have reached isotopic steady-state with local prey resources. In species where migration patterns occur on shorter timescales than tissue turnover rates, more advanced analytical approaches may be necessary for SIA-based trophic inferences (e.g. Carlisle et al. [8]).

Finally, it is important to note that our reported Δ^13^C values are for arithmetically lipid-corrected values of *δ* ^13^C for both PBFT and food tissues. Bulk *δ* ^13^C values are available in Table 3 allowing for calculation of Δ^13^C for any combination of predator/prey *δ* ^13^C values (i.e. Δ^13^C for bulk tissues can be calculated from Table 3). When applying the Δ^13^C values found here to field data, it is important that researchers use a Δ^13^C value that is based on the approach (bulk or lipid-corrected) they use for their own consumer and prey *δ* ^13^C data.

### PBFT growth

Pacific bluefin tuna growth in captivity was generally linear from 0–725 days (0.050±0.025 cm/day, Fig. 2). Variation of growth rate *k′* was highest in short-duration fish (Fig. 3). Sources of this variation may be related to fish density in the tanks (which varied over time) or differences in the acclimation time and feeding in the first days in the TRCC across individual fish, as time to first feeding can vary in captive tuna [34]. Increased growth after 0–725 days (Fig. 2) is likely due to both natural growth dynamics of PBFT [58] and increased food rations and space availability in MBA tanks. However *k′* remained generally constant throughout the experiment (*k′* = 0.0016, Fig. 3) and most fish were near, or had reached, steady-state conditions with diet before the move to MBA tanks (∼725 days, Fig. 1).

Growth-based models fit isotope turnover well (Fig. 4), though correlation coefficients were slightly lower than for time-based models (Table 4). The proportion of ^13^C and ^15^N turnover attributed to growth (i.e. dilution) versus metabolism was estimated for both tissues. As expected, metabolism accounted for the majority of turnover in liver for both isotopes (85% for ^15^N, 63% for ^13^C; Table 5), due to the high metabolic activity of liver. Interestingly, and in contrast to other studies [18], [20], metabolic processes accounted for a significant proportion of isotopic turnover in white muscle for both ^15^N (62%) and ^13^C (41%) (Table 5). Growth or dilution effects dominated isotope turnover in other species, accounting for up to 100% of turnover in some fish species [17], [18]. High metabolic influence on isotopic turnover in PBFT here is likely due to fish size and physiology. Most laboratory studies done previously have used larval or small, rapidly growing, juvenile fish (<0.5 kg) while our fish ranged from 4.5–205.4 kg (Table 1). Pacific bluefin tuna are endothermic fish and have higher metabolic rates than ectothermic fishes [42], [59], [60], [61], [62], thus PBFT use more energy per unit mass to maintain an elevated metabolic rate and less energy is available for growth relative to other species. High metabolic contributions to tissue turnover have been demonstrated in other endothermic species; for example, Carleton and Martínez del Rio [63] found high metabolic rate influenced turnover rates in endothermic birds. Endothermic physiology [20], [64], [65], [66], [67] and larger size (and subsequently lower relative growth rates) of captive PBFT than fish in other studies [37] likely account for the larger role of metabolism in isotope turnover in captive PBFT.

### Applications to field data

The power and breadth of isotopic techniques has been demonstrated in the development and application of novel predictive and statistical tools using SIA data. Dietary mixing models (e.g. MixSir [12], IsoSource [21] and SIAR [68]) generate estimates of relative proportion of dietary inputs. Isoscapes [7], when used with accurate parameters, can estimate the origin and timing of migration in animals that move between isotopically discrete regions. The results here provide the necessary parameters to perform species-specific isotopic studies on Pacific bluefin tuna.

Dietary mixing models assume that consumers are at steady-state with diet and use consumer TDF (± SD) as a model input, and some mixing models have been shown to be highly sensitive to TDF values [12], [21]. To date many studies have used the across-taxa mean of 3.4‰ for Δ^15^N in white muscle from Post et al. [11], which may in many cases be inappropriate (D. Post, pers. comm.). In this study both *δ* ^13^C and *δ* ^15^N values clearly reached steady-state conditions in both liver and white muscle (Fig. 1) so we are able to provide with confidence accurate values for TDF that can be used in genus- or species-specific isotope mixing models [5].

As highly migratory species, PBFT and other tunas can benefit from SIA studies using isoscapes [7] which allow inferences of both the origin and timing of migration. Electronic tagging has greatly increased our knowledge of movements of highly migratory species (HMS), particularly in the Pacific as a result of the Tagging of Pacific Predators (TOPP) program [30]. Electronic tags have revealed seasonally consistent migration patterns in certain species that utilize the CCLME, such as Pacific bluefin tuna that remain in the CCLME or make trans-Pacific migrations, or albacore (*Thunnus alalunga*) that either overwinter in the CCLME or in the sub-tropical gyre [30], [69], [70]. White (*Charcharodon carcharias*) and mako (*Isurus oxyrinchus*) sharks make inshore-offshore migrations, moving between highly productive ecoregions and oligotrophic areas [8], [30], [71], [72]. The obvious benefit to SIA versus electronic tagging is that isotopic values allow for retrospective inferences of movement and trophic ecology while electronic tagging data is prospective from the time of animal tagging to time of recapture. Thus electronic tags provide high resolution data on the movements of animals within the tagging ecoregion and often the ecoregions they migrate to, but cannot provide data to infer migratory origin or relative trophic ecology between ecoregions [8]. Studies that utilize the retrospective data from SIA with the prospective data from electronic tags, such as Seminoff et al. [73] in endangered leatherback sea turtles and Carlisle et al. in white sharks [8], demonstrate the power of combining these approaches.

Tunas such as bluefin that have complex migration patterns that may be population-specific require well-conceived experiments. PBFT may exhibit residential behavior or trans-Pacific-scale movements that occur with ontogeny and may be on time scales less than the time necessary for tissue-diet steady-state conditions. Therefore tuna tissues will have tissue isotopic values that represent a mixture of their recent foraging regions. The TDF and turnover values determined here could be applied to wild bluefin to estimate their migratory history. Change in δ^15^N values of wild Pacific bluefin tuna that recently migrated from Japan to the California Current was similar to observations in captive fish here (Fig. 5). While this approach needs further, rigorous analysis, it suggests that isotopic turnover in this study was similar to that in the wild. Thus for Pacific bluefin tuna in particular, stable isotope analysis may reveal recent migratory origin and timing [74]. Similar approaches have been applied to isotopic compositions of otoliths in Atlantic bluefin tuna (*Thunnus thynnus*) to discern Mediterranean- from Gulf of Mexico-spawned bluefin off the eastern US coastline [75]. Such studies will prove extremely valuable as we move towards better international management of bluefin species.

Our results also allow for general reliability estimates of data from certain isotopes and tissues. Our model fits demonstrate that ^15^N content in white muscle, for example, turns over at a much more predictable rate that does ^13^C content in liver. While several tools that use SIA data benefit from using multiple tissues, we suggest here that at least in PBFT, white muscle may provide more reliable estimates of migratory and trophic history, and while liver tissues supply useful additional information, the high variability of liver isotope values means that interpretation should be treated with care. Overall, this long-term experiment provides new parameters for isotopic studies of bluefin tunas. It demonstrates that long term laboratory studies of other pelagic animals are necessary as we move forward using stable isotope analysis to describe the movements and ecology of these important pelagic apex predators.

## Materials and Methods

### Captive Husbandry of Tunas

Juvenile Pacific bluefin tuna, *Thunnus orientalis*, were collected by hook and line off the coast of San Diego, CA, during July–September from 2000 to 2010 according to methods in Farwell [34]. These fish are born in the western Pacific and forage for a year prior to migrating to the eastern Pacific [70], [76], [77]. Bluefin are transported to the Tuna Research and Conservation Center (TRCC) for research purposes and/or display at the Monterey Bay Aquarium in Pacific Grove, CA. Size at collection was measured as curved fork length (CFL) and ranged from 62.5 to 92 cm. Tunas were held aboard the F/V *Shogun* in seawater-filled wells and subsequently transported in a 4000 L tank to the TRCC. Fish were held in three tanks at approximately 20°C (SD ±0.2°C) until they were sacrificed for research according to IACUC protocols or incurred natural mortality. Fish were fed a consistent ratio of sardines, squid, and enriched gelatin as previously described [34]. Tunas were fed thawed frozen food in the TRCC (by mass: squid 60%; sardine 31%; gelatin 9%) that have higher weighted mean *δ* ^13^C and *δ* ^15^N values (*δ* ^13^C: −17.4±0.3; *δ* ^15^N: 13.9±0.7) than white muscle (WM) and liver (LIV) tissues of juvenile year class one and two (YC1 and YC2) PBFT (*δ* ^13^C: −18.0±0.2; *δ* ^15^N: 11.8±0.2) captured off southern California. These experimental conditions allowed calculation of tissue turnover rates and tissue-specific trophic discrimination factors (TDFs) of *δ* ^13^C and *δ* ^15^N values in WM and LIV of juvenile, growing PBFT as they approached and maintained isotopic steady-state conditions with the isotopic composition of the feed. Some fish were moved as they reached large size from the TRCC facility to the Monterey Bay Aquarium (MBA) tanks, where food ratios (kg food∶kg tunas) remained the same although the quantity of food increased. Transport usually occurred after ∼700 days in the TRCC, so only the largest fish with the longest duration in captivity were subjected to conditions in MBA tanks. Tissue samples were routinely collected over the course of ten years and frozen at −20°C. Archived tissues were used opportunistically for this study.

Samples of captive food (sardine and squid WM tissues and gelatin) were sampled periodically through the years of 2010–2011 to ensure seasonal and inter-annual consistency of feed values. Squid and sardine feed are of coastal CA origin, and though *δ* ^13^C and *δ* ^15^N values in zooplankton have been shown to change on short timescales, considerable long-term stability of isotope values has been demonstrated in the California Current Ecosystem [78], [79]. Consequently the isotopic compositions of feed from previous years were assumed to be similar to those analyzed in this study. For this study, we used only tissues from Pacific bluefin that were of similar size at capture (62.5 to 75 cm) and were healthy and feeding at the time of mortality. A total of 69 PBFT were sampled for WM and 62 for LIV tissues. Liver tissue was not available for 7 of the 69 individuals analyzed, either due to sample unavailability or C∶N ratios too high for appropriate application of lipid-correction algorithms [52]. Time in captivity ranged from 1–2914 days (485±607 days).

### Isotope analysis

PBFT WM tissue was collected from the hypaxial musculature under the first dorsal fin of the animal and ∼20 cm below the skin. Liver tissue was collected from the interior of the center lobe of the liver. From sardines, a section of dorsal WM was taken; from squid, a section of mantle with the outer membrane removed. Tissues were frozen at −80°C and subsequently lyophilized and ground to a homogenous powder for isotope analysis. The *δ* ^13^C and *δ* ^15^N values of all samples were determined at the Stanford Stable Isotope Biogeochemistry Laboratory using a Thermo Finnigan Delta-Plus IRMS coupled to a Carlo Erba NA1500 Series 2 elemental analyzer via a Thermo Finnigan Conflo II interface. Replicate reference materials of either graphite NIST RM 8541 (USGS 24), acetanilide, ammonium sulfate NIST RM 8547 (IAEA N1) or glutamic acid (USGS 40) were analyzed between approximately 8 unknowns and each had a standard deviation <0.15‰. Isotope ratios are described by:
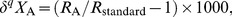
(1)where *q* is the isotope of interest, *X* is the element of interest, *A* is the tissue type (e.g. muscle or liver), *R*_A_ is the ratio of the rare to the common isotope, and *R*_standard_ is the isotope standard Air or V-PDB. Isotope values are reported as per mille (‰).

### Arithmetic corrections of *δ* ^13^C values

White muscle and liver *δ* ^13^C values were lipid-normalized based on bulk C∶N values (by mass) following calculations in Logan et al. [51] due to the ability of variable lipid content to bias *δ* ^13^C measurements [50], [51] (Table S1). We used species- and tissue-specific lipid correction factors for Atlantic bluefin tuna *Thunnus thynnus* [51]:
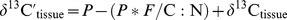
(2)where *δ* ^13^C′_tissue_ is the arithmetically-corrected *δ* ^13^C_tissue_ value, C∶N is the atomic C∶N ratio of the specific sample, and *P* and *F* are parameter constants based on measurements by Logan et al. [51]. While both chemical and lipid extractions have been shown to be effective methods to correct bias in *δ* ^13^C values based on lipid-content, arithmetic corrections preserve sample integrity and simplify sample preparation [50]. Arithmetic corrections are especially useful and more reliable when organism- and tissue-specific algorithms are available, as was the case here [50], [51]. Arithmetic corrections to tissue *δ* ^13^C values only eliminate variability of lipid content, and the subsequent variability in *δ* ^13^C, across sample types.

### Estimating turnover rate

We used an exponential fit model for two tissues (WM and LIV) and two isotope values (*δ* ^13^C and *δ* ^15^N) as used previously [19], [65], [66], [67]:
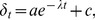
(3)where *δ_t_* is the isotope value of interest changing with time *t*, *a* and *c* are parameters derived from the best fit, and *λ* is a data-derived first-order rate constant. Parameters *a* and *c* represent important resultant parameters: *a* = isotope difference between initial and final steady-state values and *c* is the data-derived final isotope steady-state value [67]. The tissue- and isotope-specific half-life (*t*_0.5_) is then calculated:
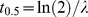
(4)for different *λ* values derived for *δ* ^15^N_WM_, *δ* ^15^N_LIV_, *δ* ^13^C_WM_, and *δ* ^13^C_LIV_. We used a modified equation from Buchheister and Latour [18] to calculate the time needed to obtain a given percentage (*α*) of complete turnover:
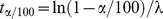
(5)where *t*_α/100_ is the time needed to attain *α*% turnover and *λ* is the data-derived first-order rate constant.

Multi-compartment models can sometimes provide better insight into turnover dynamics than first-order, one-compartment models [35], [36]. We used the reaction progress variable model of Cerling et al. [35] to evaluate whether a single-compartment model with first-order kinetics adequately described the changes in carbon and nitrogen isotopic compositions of white muscle and liver tissues in PBFT:
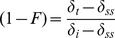
(6)where *δ_t_* is the isotopic value at time *t* during the experiment, *δ_i_* is the data-derived initial isotopic value (*c* – *a* in eqn. 3), and *δ_ss_* is the data-derived isotope final steady-state value (Table 3). A linear fit to ln(1−F) as a function of time has been shown to be consistent with a system that can be well-described by a single compartment model (35,36). Modeling the change in *δ* ^15^N and *δ* ^13^C values of white muscle and liver tissues using equation 6 indicates that a single-compartment model adequately described the results (Figure S1).

We compared our carbon turnover rates to those predicted by allometric scaling in fish reported in Weidel et al. [37]. This study showed that fish mass (g) was a strong predictor of fish carbon turnover rates (*r* ^2^ = 0.71) with the equation:
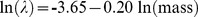
(7)We used final mass (*W_f_*) to generate a mean (± SD) value for carbon turnover in Pacific bluefin white muscle to assess the application of this equation to Pacific bluefin, and to assess whether allometric scaling of turnover rates adequately predicted the carbon turnover rates we observed in PBFT.

### Calculating TDF

We calculated trophic discrimination factor (TDF) using the difference between mean *δ* ^15^N_WM_, *δ* ^15^N_LIV_, *δ* ^13^C_WM_, and *δ* ^13^C_LIV_ values from animals that had reached steady-state with diet and the weighted mean *δ* ^15^N and *δ* ^13^C values of food, which was consistent over time. TDF values (Δ_TISSUE_) are calculated for white muscle and liver according to the equation:
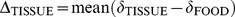
(8)where Δ_TISSUE_ represents the tissue- and isotope-specific TDF, *δ*_TISSUE_ is the nitrogen or carbon isotope value of a specific tissue for each animal that reached steady-state with diet, and *δ*_FOOD_ is an average nitrogen or carbon isotope value of the food (here squid, sardine, and gelatin supplement) arithmetically lipid-corrected [51] and weighted by the proportional mass of each item in the control diet. TDF values were calculated from isotope values from tissues in animals that had been in captivity for enough time to reach 95% turnover (*t*_0.95_, Table 3).

We compared our experimentally-derived TDF values to the Diet-Dependent Discrimination Factor algorithms reported by Caut et al. [55]. We used the fish white muscle equation from that study for ^15^N:
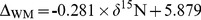
(9)and compared that theoretical TDF to our experimentally-derived value.

### Effects of growth

Fish length (CFL) was recorded at *t*_0_ and *t*_f_, and standard length (SL) estimated from CFL. Only final mass (*W_f_*) was measured directly at *t_f_*. Initial mass (*W_i_*) was estimated from SL using the equation:
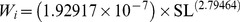
(10)from Deriso and Bayliff [80]. Relative gain in mass (*W_R_*, hereafter referred to as ‘relative growth’) was then calculated:
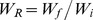
(11)where *W_f_* is the measured final mass and *W_i_* is the initial mass estimate from SL. Using the equation from Ricker [81] for *W_f_*:
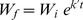
(12)where *k′* is the group specific growth-rate constant, we derive *k′*:
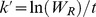
(13)and can obtain the growth rate constant *k′* for all fish using relative growth (*W_R_*) and time in captivity *t*. Hesslein et al. [14] describes the isotope value of a fish at time *t* (*δ_t_*) as:
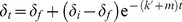
(14)where *δ_f_* is the final, or data-derived steady-state isotope value, *δ_i_* is the initial isotope value, *m* is the metabolic turnover constant, and *k′* and *t* are as previously described. This is a modification of eq. 3, where *δ_f_* = *c*, (*δ_i_*−*δ_f_*) = *a*, and (*k′*+*m*) = *λ*. Thus we calculate *λ* from eq. 3, *k*′ from eq. 13, and use eq. 14 to calculate the metabolic constant *m* for the tissue and isotope of interest. We can also calculate the amount of relative growth needed to achieve α percent turnover of *δ* ^13^C and *δ* ^15^N [18]:
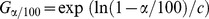
(15)and growth-based turnover can be calculated:
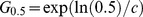
(16)where *G*_0.5_ is the growth-based half-life and *c* is the data-derived rate constant for *δ* ^13^C_WM_, *δ* ^13^C_LIV_, *δ* ^15^N_WM_, and *δ* ^15^N_LIV_. Finally, we estimate the proportion of isotopic turnover due to growth (*P_g_*) and the proportion of turnover due to metabolism (*P_m_*) as the proportion of *k′* and *m*, respectively, of the overall isotopic turnover constant *λ* [18], [81]:
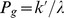
(17)

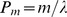
(18)We apply equations 11–18 to *δ* ^13^C and *δ* ^15^N values in PBFT WM and LIV tissues and report growth turnover constants, metabolic turnover constants, and overall estimated contribution of growth and turnover to observed isotope turnover in captive PBFT for both tissues and isotopes.

### Comparison of captive and wild *δ* ^15^N values

We plotted wild Pacific bluefin tuna data from a companion study [74] to compare turnover of wild fish that, based on size, are known to have recently arrived to the California Current from the Western Pacific Ocean [70], [74], [76], [77] to turnover of captive fish analyzed here. We used the smallest wild bluefin in the dataset (61.6 cm) as a starting size for recent migrants in the CCLME (*t* = 0) and estimated each wild bluefin tuna's residency time in the CCLME according to the growth equation from Bayliff et al. [82]:
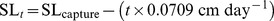
(19)and solved for *t* for each wild fish to gain an estimate of residency time in the CCS. *δ* ^15^N values of wild fish were plotted against time with captive fish to visually compare turnover of captive and wild datasets.

### Ethics Statement

All procedures used in these experiments were in accordance with Stanford University institutional animal use protocols.

## Supporting Information

Figure S1 **Reaction progress variable model (RPV) results for white muscle and liver** ***δ*** **^15^N and** ***δ*** **^13^C values in captive Pacific bluefin tuna** ***Thunnus orientalis*** **.** RPV results shown for (a) nitrogen isotopic composition of white muscle tissue, (b) carbon isotopic composition of white muscle tissue, (c) nitrogen isotopic composition of liver and (d) carbon isotopic composition of liver for PBFT showing little evidence for a curvilinear fit to the data. Results are modeled for tuna grown in the Tuna Research and Conservation Center tanks (0–725 days), where growth was linear and the greatest changes in isotopic compositions occurred. Results shown are not corrected for effects of growth on turnover; however similar conclusions are obtained for growth-corrected results. Note that results of the reaction progress variable are undefined when *δ_t_* exceeds *δ_ss_*; consequently these few data points were not included in our diagnostic analysis.
(DOC)

Table S1 **Bulk tissue C∶N ratios (by mass), bulk** ***δ*** **^13^C values, and arithmetically-corrected** ***δ*** **^13^C values (** ***δ*** **^13^C′) for white muscle (WM) and liver (LIV) tissues for all Pacific bluefin tuna used in this study.** Bulk tissue *δ* ^13^C values are arithmetically corrected using tissue-specific algorithms for Atlantic bluefin tuna *Thunnus thynnus* from Logan et al. [51]. For liver tissues, ‘ns’ indicates that liver was not sampled; ‘n/a’ indicates that C∶N value was too high for arithmetic correction.
(DOCX)
