# Supplementary figures and images for: Tissue Turnover Rates and Isotopic Trophic Discrimination Factors in the Endothermic Teleost, Pacific Bluefin Tuna (Thunnus orientalis)
Source: PLoS One. 2012 Nov 7;7(11):e49220. doi: 10.1371/journal.pone.0049220 (PMC3492276; doi:10.1371/journal.pone.0049220)

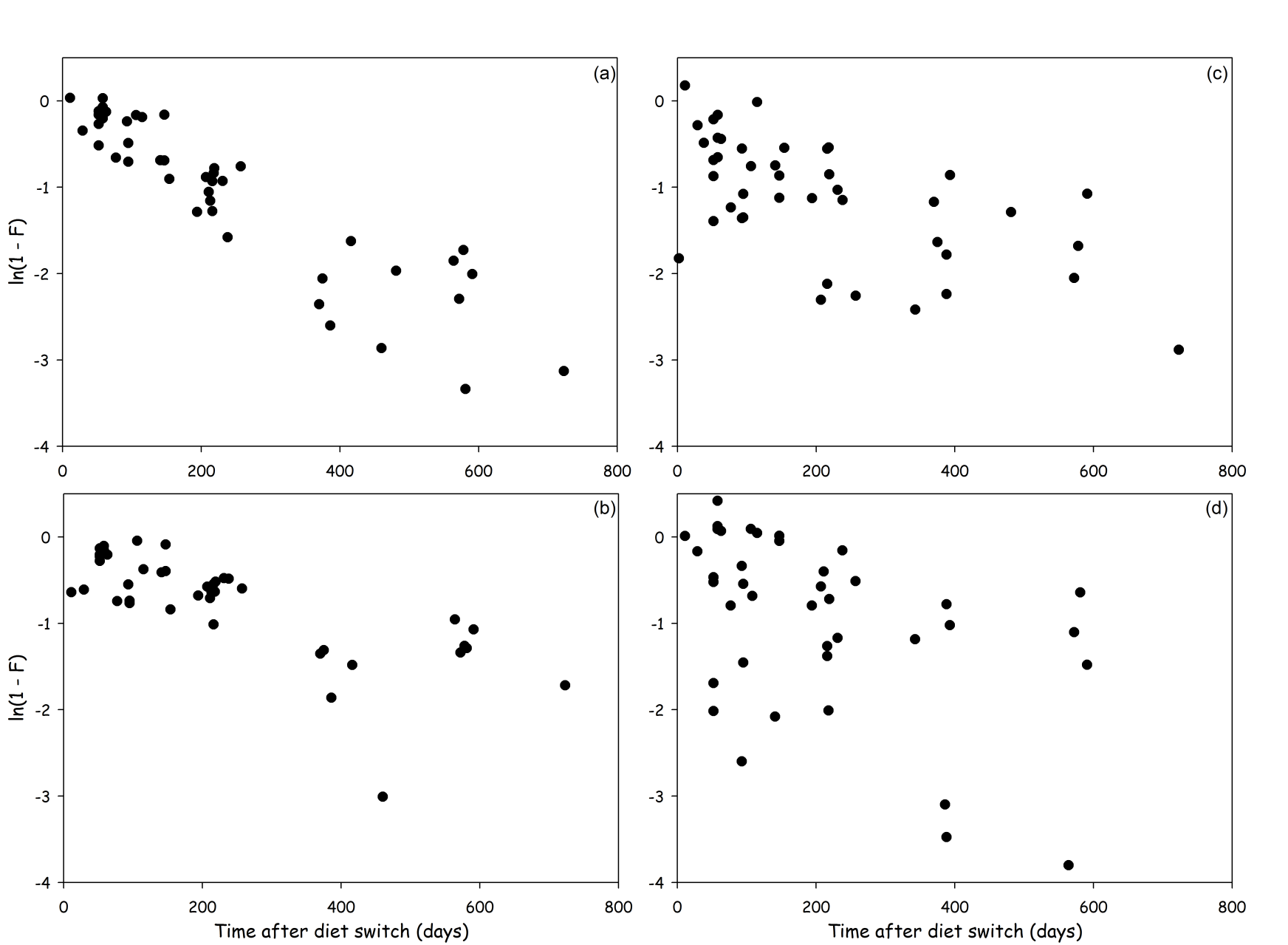
**Figure S1**

Supplement: Figure S1 — Reaction progress variable model (RPV) results for white muscle and liver δ 15N and δ 13C values in captive Pacific bluefin tuna Thunnus orientalis . RPV results shown for (a) nitrogen isotopic composition of white muscle tissue, (b) carbon isotopic composition of white muscle tissue, (c) nitrogen isotopic composition of liver and (d) carbon isotopic composition of liver for PBFT showing little evidence for a curvilinear fit to the data. Results are modeled for tuna grown in the Tuna Research and Conservation Center tanks (0–725 days), where growth was linear and the greatest changes in isotopic compositions occurred. Results shown are not corrected for effects of growth on turnover; however similar conclusions are obtained for growth-corrected results. Note that results of the reaction progress variable are undefined when δt exceeds δss; consequently these few data points were not included in our diagnostic analysis. (DOC) [file pone.0049220.s001.doc]
