# Supplementary material for: Tissue Turnover Rates and Isotopic Trophic Discrimination Factors in the Endothermic Teleost, Pacific Bluefin Tuna (Thunnus orientalis)
Source: PLoS One. 2012 Nov 7;7(11):e49220. doi: 10.1371/journal.pone.0049220 (PMC3492276; doi:10.1371/journal.pone.0049220)
Supplement: Table S1 — Bulk tissue C∶N ratios (by mass), bulk δ 13C values, and arithmetically-corrected δ 13C values ( δ 13C′) for white muscle (WM) and liver (LIV) tissues for all Pacific bluefin tuna used in this study. Bulk tissue δ 13C values are arithmetically corrected using tissue-specific algorithms for Atlantic bluefin tuna Thunnus thynnus from Logan et al. [51]. For liver tissues, ‘ns’ indicates that liver was not sampled; ‘n/a’ indicates that C∶N value was too high for arithmetic correction. (DOCX) [file pone.0049220.s002.docx]

**Table S1**

| **Sample #** | **Time in captivity (d)** | **WM C:N (by mass)** | **WM δ^13^C (‰)** | **WM δ^13^C′ (‰)** | **LIV C:N (mass)** | **LIV δ^13^C (‰)** | **LIV δ^13^C′ (‰)** |
| --- | --- | --- | --- | --- | --- | --- | --- |
| 1 | 1 | 3.6 | -18.8 | -17.8 | 9.2 | -23.6 | -18.8 |
| 2 | 1 | 4.0 | -19.5 | -17.9 | 5.0 | -21.0 | -18.8 |
| 3 | 1 | 4.2 | -20.1 | -18.2 | 11.3 | -24.7 | -19.3 |
| 4 | 2 | 3.1 | -18.4 | -18.4 | 7.4 | -19.7 | -15.7 |
| 5 | 11 | 3.1 | -17.1 | -16.9 | 3.9 | -18.6 | -17.9 |
| 6 | 29 | 3.2 | -17.3 | -17.0 | 4.2 | -18.7 | -17.7 |
| 7 | 38 | 3.2 | -16.6 | -16.3 | 4.6 | -17.7 | -16.0 |
| 8 | 52 | 3.1 | -17.8 | -17.9 | 4.9 | -18.5 | -16.5 |
| 9 | 52 | 3.0 | -17.5 | -17.7 | 4.4 | -18.7 | -17.3 |
| 10 | 52 | 3.1 | -17.6 | -17.7 | 4.6 | -18.2 | -16.5 |
| 11 | 52 | 3.1 | -17.5 | -17.6 | 4.4 | -18.7 | -17.2 |
| 12 | 58 | 3.1 | -17.9 | -17.8 | 4.0 | -19.6 | -18.8 |
| 13 | 58 | 4.0 | -19.7 | -17.9 | 3.9 | -18.7 | -18.1 |
| 14 | 58 | 3.2 | -18.1 | -17.8 | 4.1 | -19.1 | -18.1 |
| 15 | 63 | 3.2 | -17.9 | -17.7 | 4.2 | -19.1 | -18.0 |
| 16 | 77 | 3.2 | -17.1 | -16.8 | 4.2 | -18.1 | -17.0 |
| 17 | 93 | 3.1 | -17.1 | -17.1 | 4.4 | -17.8 | -16.4 |
| 18 | 93 | 3.1 | -16.8 | -16.8 | 4.2 | -18.5 | -17.4 |
| 19 | 95 | 3.1 | -16.7 | -16.6 | 4.0 | -18.0 | -17.2 |
| 20 | 95 | 3.2 | -17.0 | -16.8 | ns | — | — |
| 21 | 95 | 3.1 | -16.8 | -16.8 | 4.9 | -18.7 | -16.6 |
| 22 | 106 | 3.1 | -18.1 | -18.1 | 4.0 | -18.9 | -18.1 |
| 23 | 108 | 3.2 | -17.0 | -16.8 | 4.1 | -18.0 | -17.1 |
| 24 | 115 | 3.0 | -17.2 | -17.4 | 4.0 | -18.7 | -18.0 |
| 25 | 141 | 3.1 | -17.3 | -17.3 | 4.8 | -18.4 | -16.4 |
| 26 | 147 | 3.1 | -17.9 | -18.0 | 4.0 | -18.5 | -17.8 |
| 27 | 147 | 3.1 | -17.3 | -17.3 | 4.1 | -18.9 | -17.9 |
| 28 | 154 | 3.1 | -16.8 | -16.7 | ns | — | — |
| 29 | 194 | 3.1 | -16.8 | -16.9 | 3.8 | -17.3 | -17.0 |
| 30 | 207 | 3.5 | -18.0 | -17.0 | 4.1 | -18.1 | -17.2 |
| 31 | 211 | 3.2 | -17.0 | -16.9 | 4.2 | -18.4 | -17.4 |
| 32 | 213 | 3.2 | -17.1 | -17.0 | ns | — | — |
| 33 | 216 | 3.6 | -18.1 | -17.1 | 6.4 | -20.2 | -16.7 |
| 34 | 216 | 3.5 | -17.3 | -16.5 | 4.5 | -18.2 | -16.7 |
| 35 | 218 | 3.3 | -17.5 | -17.0 | 5.8 | -19.5 | -16.5 |
| 36 | 219 | 3.1 | -17.2 | -17.1 | 5.3 | -19.6 | -17.1 |
| 37 | 231 | 3.8 | -18.5 | -17.2 | 7.0 | -20.6 | -16.8 |
| 38 | 238 | 3.6 | -18.3 | -17.2 | 4.7 | -19.5 | -17.7 |
| 39 | 257 | 3.1 | -17.1 | -17.0 | 3.9 | -17.8 | -17.2 |
| 40 | 343 | 3.1 | -16.6 | -16.6 | 7.5 | -20.9 | -16.7 |
| 41 | 370 | 3.1 | -16.3 | -16.2 | 4.9 | -17.6 | -15.6 |
| 42 | 375 | 4.2 | -18.3 | -16.3 | 8.0 | -20.4 | -16.0 |
| 43 | 386 | 3.3 | -16.4 | -16.0 | 4.4 | -17.7 | -16.3 |
| 44 | 388 | 3.1 | -16.5 | -16.4 | 5.0 | -18.5 | -16.3 |
| 45 | 393 | 3.1 | -17.0 | -17.0 | 4.3 | -18.0 | -16.8 |
| 46 | 416 | 3.2 | -16.5 | -16.2 | 5.7 | -18.1 | -15.2 |
| 47 | 460 | 3.1 | -15.8 | -15.7 | 4.5 | -17.6 | -16.0 |
| 48 | 481 | 3.2 | -15.7 | -15.4 | 4.7 | -17.9 | -16.1 |
| 49 | 564 | 3.1 | -16.6 | -16.6 | 4.3 | -17.6 | -16.3 |
| 50 | 572 | 3.9 | -17.9 | -16.3 | 5.5 | -19.5 | -16.8 |
| 51 | 578 | 3.3 | -16.7 | -16.3 | 5.8 | -18.4 | -15.4 |
| 52 | 581 | 3.0 | -16.2 | -16.3 | 3.7 | -17.3 | -17.1 |
| 53 | 591 | 3.5 | -17.4 | -16.5 | 4.5 | -18.2 | -16.6 |
| 54 | 698 | 7.5 | -20.3 | -15.9 | 5.4 | -17.7 | -15.1 |
| 55 | 704 | 3.2 | -15.6 | -15.5 | ns | — | — |
| 56 | 723 | 3.1 | -16.1 | -16.0 | 4.6 | -17.7 | -16.0 |
| 57 | 828 | 9.1 | -21.4 | -16.5 | ns | — | — |
| 58 | 1054 | 3.1 | -16.2 | -16.3 | 4.6 | -18.6 | -16.9 |
| 59 | 1065 | 4.2 | -17.8 | -15.8 | 9.3 | -20.7 | -15.8 |
| 60 | 1189 | 3.5 | -15.9 | -15.0 | ns | — | — |
| 61 | 1225 | 6.1 | -19.2 | -15.4 | 11.9 | -21.4 | -15.9 |
| 62 | 1461 | 4.0 | -17.2 | -15.4 | 4.2 | -17.4 | -16.3 |
| 63 | 1483 | 5.9 | -19.1 | -15.6 | 4.7 | -18.3 | -16.5 |
| 64 | 1527 | 3.9 | -16.9 | -15.4 | 10.9 | -21.5 | -16.2 |
| 65 | 1666 | 3.2 | -15.4 | -15.3 | 4.5 | -18.0 | -16.4 |
| 66 | 1897 | 7.9 | -20.4 | -15.9 | 12.1 | -22.2 | -16.6 |
| 67 | 1947 | 7.2 | -19.8 | -15.5 | >13 | -22.2 | n/a |
| 68 | 2289 | 3.4 | -16.1 | -15.5 | 9.0 | -20.7 | -15.9 |
| 69 | 2914 | 3.5 | -16.8 | -15.9 | ns | — | — |
